# An acetone extract of *Clausena anisata* may be a potential control agent for flies encountered in cutaneous myiasis

**DOI:** 10.4102/ojvr.v83i1.1045

**Published:** 2016-05-24

**Authors:** Lillian Mukandiwa, Jacobus N. Eloff, Donald R. Sibanda, Vinny Naidoo

**Affiliations:** 1 Department of Paraclinical Sciences, University of Pretoria, South Africa; 2 Mpumalanga Veterinary Services, Siyabuswa, South Africa

## Abstract

*Clausena anisata* is a medicinal plant used traditionally to treat myiasis and as an insect repellent by various communities. We have previously demonstrated the effects of *C. anisata* extracts on blowfly feeding and development in our laboratory. The impact of *C. anisata* leaf extracts on populations of different fly species on farms in Mpumalanga, South Africa was investigated in this study under field conditions. Flies were exposed to liver baits treated with acetone leaf extracts of *C*. *anisata* (150 mg/mL). Fly numbers and composition on two farms, with and without *C. anisata* treated liver, were compared during a period of 12 weeks when fly populations were expected to be high. Observations were made on fly behaviour and development, adult sizes and numbers. The flies exposed to liver treated with the leaf extract of *C. anisata* had a decreased rate of development, prolonged larval period, smaller body sizes and more sluggish behaviour compared to those subjected to the control treatment. No significant differences were, however, found between the numbers and sizes of flies on the treated and on the control farm, which was most likely due to the limited nature of the baiting programme we followed. The effects of *C*. *anisata* extracts on blowfly behaviour and development observed in previous laboratory studies were confirmed in this field evaluation. Although the extracts did not have a significant effect on the overall population size in this experiment, we believe that the *C. anisata* leaf extract could be useful in integrated pest management based on its effect on larval development. In addition, species such as *Lucilia cuprina* and *Chrysomya marginalis* seemed to have been repelled by the *C. anisata* treated liver; as a result, further work should explore this aspect and how it can be used for the protection of animals.

## Introduction

Myiasis, the infestation of live vertebrate animals with fly larvae, causes discomfort, loss in production, reproduction problems, blindness, lameness and even death in production animals (Sotiraki & Hall 2012). Although myiasis has been recognised as a major disease from ancient times (Sherman, Hall & Thomas 2000), in modern times the disease is still poorly controlled in the animal production industry of many countries. This leads to severe economic losses and animal deaths (Sotiraki & Hall 2012; Wall 2012). Economic losses occur through abortions, decreased milk production, lower production, lower fertility, poor hide quality, muscle damage and even death from toxicity or secondary infections. There are 18 different genera of flies that may be involved in cutaneous myiasis. They belong to seven families, namely Calliphoridae, Sarcophagidae, Muscidae, Phoridae, Cuterebridae, Gasterophilidae and Oestridae (Hall 1991). Species of flies in the Calliphoridae, Sarcophagidae and Muscidae families are the main cause of cutaneous myiasis (Hall 1991).

Control of myiasis has largely relied on an integrated approach incorporating insecticides, husbandry practices (such as shearing, crutching, tail docking) (Phillips 2009), fly trapping (Urech *et al*. 2009; Wall 2012) and, in Australia, mulesing (Tellam & Bowles 1997). Despite the integrated approach, a sustained reliance on the use of insecticides over many years has led to the inevitable development of blowfly populations resistant to insecticides (Heath & Levot 2015). Other concerns with chemical compounds include the potential to cause human or animal toxicity. Residues left in wool released in effluent during scoring may also lead to environmental contamination. Consequently, there are increasing calls for the removal of certain blowfly control products from the international market, providing the impetus for non-insecticidal control methods (Bates 2012).

The fly population control method has been advocated as an alternative means of controlling myiasis (Knipling 1979). The advantage of this system is that the control measure decreases the number of adult flies in the environment without having to rely on the exposure of the animal and wool to high concentrations of ectoparasiticides. An example of an effective system is the LuciTrap system (Bioglobal Ltd, Australia), which has been effectively used to reduce fly densities and strike incidence in Australia (Ward & Farrell 2003) and South Africa (Scholtz *et al*. 2000). The LuciTrap system consists of a translucent bucket made from tough ultraviolet-stabilised plastic and a removable lid with a flat surface, entrance cones that allow the sheep blowfly to enter but not leave the trap, and a bottle with the chemical attractant (LuciLure) (Levot 2009). The attractant consists of chemicals designed to mimic the odours of primary food sources of the sheep blowfly: fleece rot, animal carcasses, urine and faeces.

*Clausena anisata* (Willd.) Hook. f. ex. Benth., belonging to the Rutaceae family, is used for the treatment of myiasis in some communities in Zimbabwe (Chavunduka 1976) as a maggot-expelling agent. In Ethiopia, the leaves of this plant are used to repel houseflies (Karunamoorthi & Husen 2012). It is also used as a repellent against various pests in different countries (Boeke *et al*. 2004; Mavundza *et al*. 2011; Ndomo *et al*. 2008). Our previous *in vitro* studies on the effect of the extracts of *C. anisata* on the blowfly larvae established that extracts of *C. anisata* deterred the second and third instar larvae of blowflies *Lucilia cuprina* and *Chrysomya marginalis* from feeding, resulting in lower pupae weights and smaller emerging adult flies (Mukandiwa, Eloff & Naidoo 2012a; Mukandiwa *et al.* 2012b). Furthermore, a pyranocoumarin, seselin, isolated from the plant was identified as one of the compounds that deter blowfly larval feeding (Mukandiwa *et al*. 2013). In this study we explored the possibility of suppressing populations by attraction and inhibition of the life-cycle through the use of traps baited with ox-liver mixed with a plant insecticidal extract. We evaluated the efficacy of liver treated with *C. anisata* extracts against myiasis-causing flies in field trials on farms in the Mpumalanga province, South Africa.

## Materials and methods

### Study sites

Fly populations were monitored at two farms in KwaMhlanga, 90 km north-east of Pretoria, South Africa. This area was selected based on the high prevalence of myiasis in the summer months, with in the order of 60 cases occurring per month (Area veterinarian, personal communication). Farm 1 served as a control baited with liver treated with the solvent only, whereas farm two was baited with liver treated with *C. anisata*. The farms were more than 10 km apart (treated farm: GPS coordinates 28°39’51”E, 25°21’17” and control farm GPS coordinates 28°44’48’’E, 25°33’5”) and were matched with regard to grazing environment; sheep breed, stocking density, flock management and climate. Both farms consisted of about 850 ha of predominantly natural pasture on which 100 sheep, 300 cattle and 80 goats are grazed. The migration of flies between the farms was eliminated as a factor because adult flies will not normally travel more than 3 km during their lifespan (Gleeson & Heath 1997).

### Fly monitoring

Fly abundance was monitored between December 2011 and April 2012 using Redtop flycatchers^®^. Two traps were hung 100 m away from the sheep night-camps, 300 m apart, at a height of 1.2 m above the ground on trees as per the manufacturer’s instructions. The initial trapping (which included three trappings on separate occasions) was done at the beginning of the study to establish the fly numbers on the farms. The counts from the three trappings were averaged and the average counts served as the starting fly numbers in the study areas; thereafter, fly trapping was done every 4 weeks. Each trapping lasted 48 hours and made use of rotten ox-liver as the bait. Ox-liver is a good attractant of flies encountered in myiasis (Blackwell *et al*. 1997; Parker & Welch 1991). Following each trapping, all adult Diptera were counted, measured in size and identified according to the descriptions given by Howell, Walker and Nevill (1978).

### Collection and preparation of plant material

The leaves of *C. anisata* (Willd.) Hook. f. ex. Benth. were collected in autumn from the National Botanical Garden, Pretoria, South Africa and dried at room temperature in a well-ventilated room. The plant species was identified by tree name tags and authenticated by the guide at the National Botanical Garden. The voucher specimen of the plant species, numbered PMDN317, is kept at the Medicinal Plant Collection Herbarium of the Department of Paraclinical Sciences, University of Pretoria, South Africa. Collection, drying and storage guidelines of the plant material followed were as outlined by McGaw and Eloff (2010). Dried and powdered leaves of *C. anisata* (437 g) were extracted with acetone (5 L) at room temperature by continuous agitation on an orbital shaker (Labotec^®^, model 202, South Africa) for 6 h. The mixture was filtered and the solvent removed under reduced pressure at low temperature (40 °C – 50 °C) with a rotary evaporator. The extraction process was repeated twice and extracts combined to give 37.9 g of dry acetone extract. The dried extract was reconstituted in acetone to make a 150 mg/mL stock of extract, which was used for the assays. The whole process was repeated when more extract was needed.

### Exposing flies to plant extract

**FIGURE 1:**
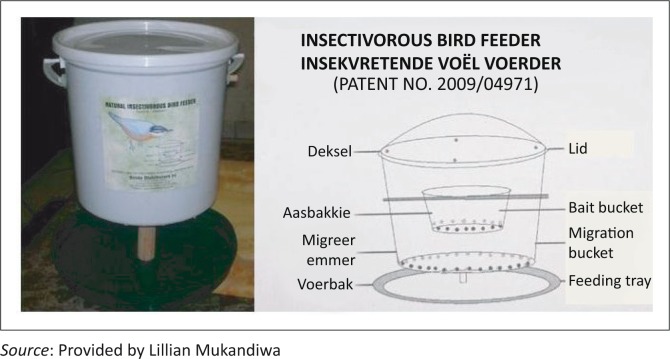
The Insectivorous Bird Feeder^®^.

After the initial trapping, Farm 2 was exposed for 4 weeks to ox-liver treated with a 150 mg/mL acetone extract of *C. anisata*, placed in the Insectivorous Bird Feeders^®^ (Stride Distributors CC, South Africa) (Figure 1). The concentration of extract was selected based on previous laboratory studies in which it was the most effective against blowfly larvae (Mukandiwa *et al.* 2012b). The feeder consists of a bait bucket with a perforated bottom inside a migration bucket, also with a perforated bottom, over a tray. Ideally, the feeding larvae migrate and drop into the feeding tray, where they can be eaten by birds. For this study the feeders were modified so that the bottom of the migration bucket was sealed to prevent the larvae from crawling out into the feeding tray of the feeder. The larvae fed on the bait in the smaller bucket until they crawled out, of their own accord, into the migration bucket, which contained wood shavings to allow for growth and development into subsequent stages.

A mixture of 100 g of crushed ox-liver and 5 mL of plant extract (150 mg/mL) was placed in the bait bucket of the feeder (*n* = 10) around the sheep night-camps, 1.2 m above the ground. The feeders were left open for an hour in open space to enable the acetone to evaporate and then the lids were placed on top of the feeders to keep the rain out. Two holes were made on either side of the feeders to allow gravid female flies to enter and lay eggs on the liver extract mixture so that the emerging larvae would become exposed to the plant extract. The feeders were inspected every 2 days and the insecticide mixture replaced every 6 days. After the 4 weeks of baiting, trapping was done with the Redtop flycatcher^®^ for 48 hours for fly number quantification (as described above) prior to another 5 weeks of exposing flies to the baited ox-liver. Farm 1, which served as the control, was exposed to the same system as above with the exception that pure acetone was used in the bait (solvent control).

At each inspection of the feeders (every 2 days) we checked for the presence of fly eggs on the baits, and larval growth, movement and feeding activity were visually evaluated. For each group of pupae, 50 normal-looking pupae were taken to the laboratory for the emerging flies to be assessed.

### Statistical analysis

The fly size data were evaluated by a non-parametric *t*-test for difference from the control group in SPSS 20 (IBM) as the counts were not normally distributed.

### Sensory evaluation of the extract

A sensory evaluation of the extract was conducted to determine the acceptability of the extract for use. The participants in this evaluation included farmers, animal health personnel (veterinarians, veterinary nurses and animal health technicians) and researchers in ethnoveterinary studies. The respondents were blinded as to what the sample was and were asked to comment on the smell. The respondents were asked to rate the extract on a scale of: very bad, bad, neutral, nice and very nice. A total of 50 respondents were used. The reason for undertaking this analysis was to ensure that the extract would be aesthetically acceptable to people who would have to work with it.

## Results

### Fly populations

The flies caught in the Redtop flycatchers^®^ were composed of blowflies *Lucilia cuprina*, *Chrysomya albiceps* and *C. marginalis*, the fleshfly, *Sarcophaga haemorrhoidalis*, and the housefly, *Musca domestica*. The changes in the populations of flies by species over time are presented in Table 1, whereas the changes in fly size are presented in Table 2. The sizes of *S. haemorrhoidalis* on the treated farm and the control farm were not significantly different (*p* = 0.362) at the beginning of the study. However, as the study progressed, the flies on the treated farm were significantly smaller (*p* = 0.02 week 4; *p < *0.00, week 8; *p* = 0.01 week 12) than those on the control farm at any given point in time. There was no particular pattern of change in size with the other fly species (Table 2).

**TABLE 1:** Number of flies over time on both the treated farm and the control farms.

Trapping	*Chrysomya albiceps*		*Chrysomya marginalis*		*Lucilia cuprina*		*Sarcophaga haemorrhoidalis*		*Musca domestica*	
				
	Treated	Control	Treated	Control	Treated	Control	Treated	Control	Treated	Control
13/01/2012	133	120	167	180	260	198	79	51	254	331
18/02/2012†	1710	180	2730	220	1560	314	87	35	670	181
23/03/2012	102	100	133	110	101	192	13	18	87	103
06/04/2012	38	17	12	33	50	71	5	8	53	74

† Trapping was done 2 weeks after a cow died on the farm and was left in the open for 3 days on the treatment farm.

**TABLE 2:** Sizes (mean ± s.d.) (mm) of the different fly species captured from the treated farm during a 12-week period.

Fly species	Time (weeks)				

	Treatment	0	4	8	12
*Chrysomya marginalis*	Test	12.2 ± 0.5^a^	12.5 ± 0.4^b^	11.7 ± 0.5^a^	10.7 ± 0.4^a^
	Control	12.2 ± 0.5^a^	12.0 ± 0.6^a^	11.8 ± 0.3^a^	11.8 ± 0.8^b^
*Chrysomya albiceps*	Test	12.0 ± 0.3^a^	12.2 ± 0.6^a^	10.7 ± 0.6^a^	11.8 ± 0.5^a^
	Control	12.1 ± 0.5^a^	12.2 ± 0.8^a^	11.1 ± 0.4^b^	11.3 ± 0.6^a^
*Lucilia cuprina*	Test	10.6 ± 0.4^b^	11.0 ± 0.4^b^	9.0 ± 0.8^a^	7.8 ± 0.7^a^
	Control	10.0 ± 0.5^a^	10.5 ± 0.6^a^	9.0 ± 0.6^a^	9.0 ± 0.9^b^
*Musca domestica*	Test	8.0 ± 0.3^a^	8.1 ± 0.3^a^	7.8 ± 0.4^a^	7.8 ± 0.5^a^
	Control	8.0 ± 0.8^a^	8.0 ± 0.0^a^	8.2 ± 0.4^b^	8.0 ± 0.5^a^
*Sarcophaga haemorrhoidalis*	Test	14.8 ± 0.3^a^	15.0 ± 0.0^b^	13.0 ± 0.6^a^	13.1 ± 0.5^a^
	control	14.6 ± 0.5^a^	14.8 ± 0.3^a^	15.0 ± 0.2^b^	14.5 ± 0.6^b^

a,b For each fly type, at a given point in time, the test and control means with different superscripts are significantly different (*p* < 0.05).

### Observations

**FIGURE 2:**
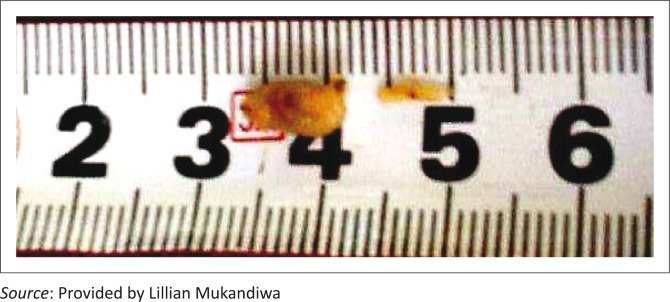
Illustration of the different size of 4-day-old larvae from the control farm and the treated farm, from left to right.

Flies were attracted to the baits of both farms and laid eggs. From the second day, according to inspections and by judging the stages of larvae observed in the baits after exposure, the eggs hatched within the mean standard period of 8–24 hours. However, by day 4 after hatching, all larvae, irrespective of species, that fed on the liver treated with *C. anisata* extract remained small, both in length and thickness, compared to the larvae that fed on liver treated with acetone only (Figure 2). By day 4, fly larvae on the control farm (Farm 1) were very active and began migrating into the migration bucket containing wood shavings (Figure 1); pupation was observed on day 6. By day 14, 90% of the pupae had hatched. However, on the treated Farm 2, the larvae were less active than the control group and more than 95% of them failed to crawl into the migration bucket, pupating instead in the bait bucket.

The larval stage was also prolonged for all the fly species that were on the treated liver, with the total period from hatching to pupation lasting 17–21 days. The larval stages lasted 4–6 days in the life-cycles of all the fly species encountered in this study, under the weather conditions in which this study was undertaken. A relatively small number, approximately 20%, of dead larvae were observed on the liver treated with the *C. anisata* extract. However, the dead larvae could not be counted as this could have disturbed the rest of the larvae.

**FIGURE 3:**
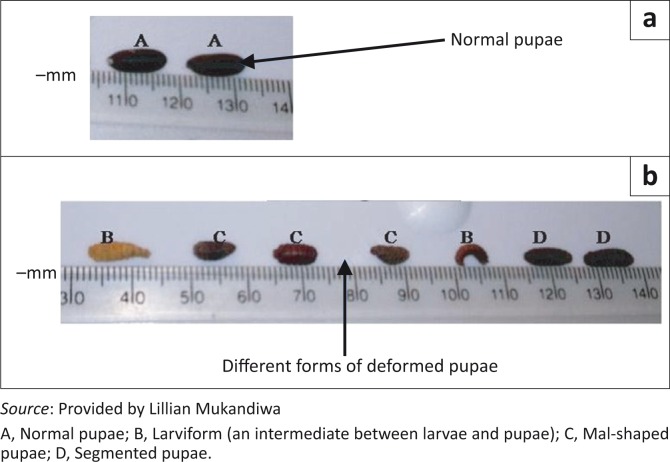
(a, b) Different forms of abnormal pupae emerging from the test farm (B–D) compared to the normal pupae from the control farm (A).

The majority of the emerging pupae (90%) from the treated groups appeared normal, and 75% of these pupae hatched after 2 weeks of pupation (days 35–42). In the control group, all the pupae hatched within 6–7 days of pupation (days 13–14). Abnormal pupae were observed on the treated farm (Figure 3).

**FIGURE 4:**
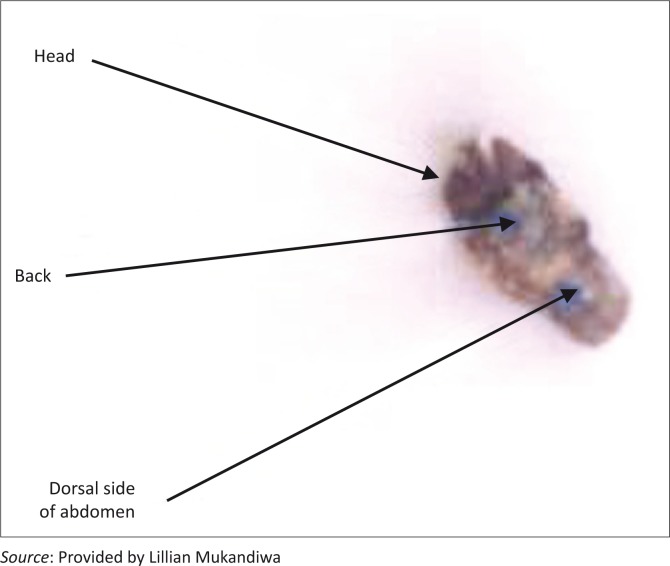
Half-developed flies from the pupae that failed to hatch naturally.

Of the 200 ± 5 pupae collected for laboratory assessment, 75% from the treated farm eclosed, yielding only the fleshfly, *S. haemorrhoidalis*; the rest of the pupae did not eclose. All the pupae from the control farm hatched, giving a mixed population of flies consisting of blowflies (77%), fleshflies (4%) and houseflies (19%). When the pupae from Farm 2 that failed to hatch were cracked open, by holding the pupae between the forefinger and thumb and gently applying pressure, half-developed flies were found that had all their appendages (head, abdomen and legs) except the wings (Figure 4).

To ascertain whether the absence of blowflies in the samples taken for laboratory assessment from the treated farm was due to the extract and not an artefact, untreated liver was hung around the sheep camps. The eggs laid were taken to the lab to develop into subsequent stages and 93% of the emerging flies were blowflies, *Lucilia* and *Chrysomya* spp.

#### Sensory evaluation of extract

**FIGURE 5:**
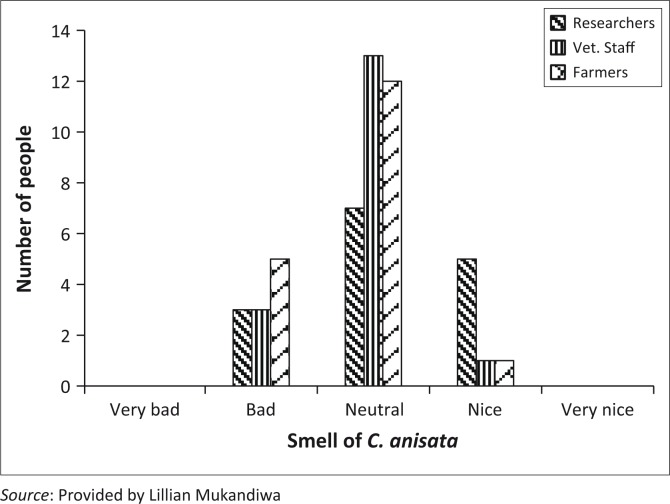
Summary of results on the sensory evaluation of the *Clausena anisata* extract.

The majority of the respondents (64%) found the smell of the extract to be neutral (neither bad nor nice), with none of the respondents rating it very bad or very nice (Figure 5).

## Discussion

One of the major findings of this study was the prolonged time of 17–21 days taken for the baited larvae to pupate in comparison to 4–6 days for the control larvae. This is an important result, as the prolongation of the life-cycle increases the chances of death in the environment. Ultimately this would reduce the total population, as the total number of life-cycles in one breeding season would be reduced.

The data from the monitoring trappings indicated the presence of a mixed population of *Lucilia*, *Chrysomya*, *Sarcophaga* and *Musca* species, but surprisingly only *S. haemorrhoidalis* emerged from the pupae collected from the treated farm. When untreated liver was hung around the sheep camps on the treated farm, the flies that emerged from the collected eggs were largely *Lucilia cuprina, Chrysomya albiceps* and *C. marginalis*. This suggests that either the *Lucilia*, *Chrysomya* and *Musca* species were more susceptible to the extract and did not survive to the adult stage, or they avoided the baits. The last option is most plausible, as the fly numbers and the size of the adult *Lucilia*, *Chrysomya* and *Muscidae* species did not significantly change as the study progressed, as was observed with *S. haemorrhoidalis*. In previous studies under laboratory conditions the *Lucilia* and *Chrysomya* species laid eggs on the treated baits (Mukandiwa *et al*. 2012b). The contradictory results may be because the flies had no other options as they were kept in a cage with only this site for oviposition. In a different setup where there are alternatives, the flies will avoid the *C. anisata* treated baits, suggesting the possibility that this extract may have potential benefits as a repellent that can be used on the animal as a wound dressing to keep blowflies away. This would also validate the traditional use in which the leaves are packed onto wounds to expel maggots (Chavunduka 1976) and probably to keep the flies away. We have previously established the safety of *C. anisata* leaf extracts in a cytotoxicity study (Mukandiwa *et al*. 2012b). However, further studies will need to be conducted *in vivo* to establish the efficacy and safety of the *C. anisata* extract as a wound dressing.

If the argument above holds that the blowflies were repelled, it does not agree with earlier studies that evaluated the extract against the blowflies *L*. *cuprina* and *C. marginalis* (Mukandiwa *et al*. 2012a, 2012b) under laboratory conditions. However, some conclusions can be drawn. The extract had a feeding deterrence effect on different fly species as the larvae on treated meat were smaller than on the control group both in the laboratory and in current studies. The extract also prolonged the pre-puparium stage in different fly species. Therefore the effects of the acetone extract of *C. anisata* with regard to reduced larval sizes and the prolonged pre-puparium stage, although for different fly species, were the same under both laboratory and field conditions.

At present, the mechanism of action of the extracts is unknown. However, based on the effects seen, 2 explanations are plausible, that is, the presence of feeding deterrents or the presence of juvenile hormone mimics. Plant-derived compounds have been shown to affect the feeding and diet-selection behaviour of the larvae of blowflies (Green, Simmonds & Blaney 2002; Mukandiwa *et al*. 2012a, 2012b). Some are toxic whereas others act as feeding deterrents; for example, *Phormia regina* larvae avoided diets containing 10 ppm and 100 ppm azadirachtin and 10 ppm pyrethrum extract (Green *et al*. 2004). The alkaloids arecoline, caffeine, quinine and nicotine, among others, reduce food consumption in blowfly larvae, resulting in reduced weights of the larvae (Green *et al*. 2002). *Clausena anisata* contains various alkaloids: atanisatin, clausanitin clausenol, clausenine girinimbine, heptaphylline, 3-methylcarbazole, 1-methyl-3,4-dimethoxy-2-quinolone, 3-formyl-1-hydroxycarbazole, murrayamine-A and ekeberginine furanoclausamine A and B, clausamine A-H, mukonal, glycosinine, mukonidine and clausine F clausanitine and mupamine (Ojewole 2002). Whereas the effects of each alkaloid listed here have not been established in laboratory studies, this class of compounds is known to act as feeding deterrents to insects and herbivores (War *et al*. 2012) In addition, *C*. *anisata* contains the coumarins imperatorin and xanthoxyletin, which have an anti-feeding effect on insects (Gebreyesus & Chapya 1983). We also isolated the pyranocoumarin, seselin (2H,8H-Benzo[1,2-b:3,4-b’]dipyran-2-one,8,8-dimethyl), from the *C. anisata* extract as the feeding deterrent (anorexogenic agent) against blowfly larvae (Mukandiwa *et al*. 2013).

The other plausible and more likely reason for the prolonged life-cycle, 21 days in comparison to the 4–6 days of the control, may be more physiologically based, as plants are known to produce insect juvenile hormones (Bede & Tobe 2000). The major function of juvenile hormone is the maintenance of the larval status or the so-called juvenilising effect (Dhadialla, Retnakaran & Smagghe 2005). The plant extract had effects similar to other insect growth regulators (IGRs), which include reduced development and a prolonged larval period, a smaller body size at a given time compared to the control, and sluggish behaviour, delayed pupation and a reduced eclosion rate of pupae and adults. The IGRs include the juvenile hormone analogues, ecdysone agonists and inhibitors and chitin synthesis inhibitors. Ultimately, IGRs control insect populations (Mondal & Parween 2000), albeit over a longer period of time. Previous studies showed that IGRs caused a decline in populations of the German cockroach (*Blattella germanica* L.) in 3–4 months after the start of baiting or spraying treatment, with complete eradication after 12 months (Mosson *et al*. 1995). Our study period may therefore have been too short for the extract to begin to have effects on the total fly populations. This is supported by the lack of a clear pattern in the fly populations over our study.

The other aim of this study was to ascertain the feasibility of using this plant extract in the field. The two major concerns were the possible unpleasant smell of the extract and chemical instability in the environment. For the first characteristic, the smell of the plant extract was scored by different individuals as being acceptable. This was an important finding, because the leaves of *C. anisata*, from which the extract was derived, are known to be densely dotted with glands and have a strong scent when crushed. The scent is highly unpleasant, characteristic of horse urine as suggested by the common Afrikaans name, *Perdepis* (horse urine) (Schmidt, Lotter & McCleland 2002). However, for this study the acetone leaf extract was rated by 64% as having a neutral smell, which implies either that the compounds that give *C*. *anisata* leaves their characteristic smell are not present in a high enough concentration in the acetone extract or that they were masked by other compounds. Regarding our concerns about its stability, the extract proved to remain constantly effective despite being changed every 5 days. In the laboratory studies the larvae were exposed to the extract for 48 hours only.

Successful fly population control requires an integrated approach that would include removal of other food sources in the environment. This was clearly evident in this study, as the unscheduled death of a cow led to a massive increase in the number of flies trapped 2 weeks later, despite baits still having their desired effect.

## Conclusion

The effect on the larval growth and pupal shape make this extract a candidate for further evaluation in the search for new fly-control products. A longer study period is necessary to establish conclusively the effect of the extract on the total population of flies in the environment. Furthermore, the extract seemed to repel the blowflies from the baits, suggesting that it may be used as a topical product on animals to repel blowflies.
